# Personalized barrier recovery induced by *Lactobacillus helveticus* SBT2171 in an In Vitro model of ulcerative colitis-derived fecal supernatants

**DOI:** 10.1186/s12906-026-05416-0

**Published:** 2026-05-23

**Authors:** Melika Khademi, Nesa Kazemifard, Mohadese Fathi, Maryam Farmani, Shaghayegh Baradaran Ghavami, Binazir Khanabadi, Shabnam Shahrokh

**Affiliations:** 1 Gastroenterology and Liver Diseases Research Center, Research Institute for Gastroenterology and Liver Diseases, Shahid Beheshti University of Medical Sciences, Tehran, Iran; 2 Department of Hematology and Blood Banking, School of Allied Medical Sciences, Shahid Beheshti University of Medical Sciences, Tehran, Iran

**Keywords:** Inflammatory bowel disease, IBD, Epithelial barrier, *Lactobacillus helveticus*, Probiotics, Personalized medicine

## Abstract

**Background:**

Disruption of the intestinal epithelial barrier is a hallmark of inflammatory bowel disease (IBD). Although probiotics and postbiotics are increasingly recognized as potential therapeutic approaches, treatment efficacy is often influenced by patient-specific variability.

**Methods:**

In this study, intestinal inflammation was modeled by exposing HT-29 cells to fecal supernatants (FS) derived from three patients with ulcerative colitis (UC). The expression of pro-inflammatory (*IL-8*) and barrier-associated (*Claudin-1*, *MUC2*) genes was assessed by real-time PCR. Subsequently, the restorative effects of different preparations of *Lactobacillus helveticus* SBT2171, including live bacteria, cell-free supernatant, and debris-containing supernatant, were evaluated at 24 and 48 h.

**Results:**

FS exposure significantly upregulated *IL-8* expression while downregulating *Claudin-1* and *MUC2*, thereby recapitulating IBD-associated epithelial disruption. Treatment with *L. helveticus* preparations partially restored barrier-related gene expression in a patient- and treatment-dependent manner. Live bacteria induced transient or sustained *Claudin-1* upregulation depending on the FS source, whereas the cell-free supernatant demonstrated broader and more durable restorative effects across distinct inflammatory contexts. In contrast, debris-containing preparations exerted limited or even suppressive effects, particularly on *MUC2* expression.

**Conclusions:**

These findings demonstrate that the barrier-restorative effects of *L. helveticus in vitro* is not uniform but shaped by the unique inflammatory and microbial environment of each patient. Collectively, our results support the potential of postbiotic formulations as safer and effective modulators of epithelial barrier repair and underscore the need for personalized strategies in microbiota-based therapies for IBD.

## Background

 The intestinal epithelial barrier plays a vital role in maintaining gastrointestinal homeostasis by serving as a dynamic and selective interface between the host and the gut microbiota. This complex structure comprises various epithelial cells, including mucus-secreting goblet cells, Paneth cells, and enterocytes, as well as intercellular adhesion components such as tight junctions (TJs), adherens junctions (AJs), and desmosomes [1–3]. By interacting with the gut microbiota, immune cells, and exosomes, the mucosal barrier effectively reinforces the separation between the intestinal lumen and the external environment, thereby preserving intestinal integrity. When the integrity of this barrier is compromised, intestinal permeability increases, allowing luminal bacteria and antigens to penetrate the epithelial layer and interact with immune cells in the underlying mucosa, a condition known as “leaky gut” [4]. This translocation leads to an exaggerated immune response, which results in chronic inflammation and tissue injury a hallmark of inflammatory bowel disease (IBD). The disruption of tight junction proteins and reduction in mucus-secreting goblet cells further weakens the barrier, creating a cycle of persistent immune activation and epithelial damage [5, 6].

IBD, including Crohn’s disease (CD) and ulcerative colitis (UC), comprises a group of chronic, relapsing inflammatory disorders of the gastrointestinal tract. Its pathogenesis is multifactorial, involving genetic susceptibility, environmental influences (particularly those affecting the gut microbiome), and barrier dysfunction. One of the well-established pathological findings in both CD and UC is the diminished number of goblet cells, which leads to reduced mucin production and increased exposure of epithelial surfaces to the gut microbiota [6–8]. Numerous studies report altered gut microbiota composition and diversity in the fecal supernatant of IBD patients compared to healthy controls [9]. Yet, no specific bacterial species has been consistently linked to IBD, and the role of microbial biomolecules remains unclear. Commensal bacteria may either promote inflammation or suppress it through regulatory mechanisms, with their loss reducing immune control [10].

Given the central role of epithelial integrity in IBD, therapies aimed at restoring the mucosal barrier have garnered considerable interest. Probiotics, defined as live microorganisms that provide health benefits, have been shown to support intestinal homeostasis by enhancing TJ integrity, increasing mucin expression, modulating immune responses, and inhibiting pathogen adhesion [11, 12]. Specifically, anaerobic bacteria such as *Lactobacillus* and *Bifidobacterium* can adhere to the intestinal epithelium and form a protective pellicle, thereby limiting the access of harmful bacteria [13]. Among these, *Lactobacillus helveticus*, a thermophilic and homofermentative lactic acid bacterium, is widely utilized in dairy fermentation, particularly for producing cheese and fermented milk. This species is distinguished by its unique capacity to metabolize galactose, rapid acidification rate, and significant proteolytic activity, setting it apart from other commonly used starter cultures in the dairy industry [14]. Similarly, postbiotics, which are non-viable microbial products like inactivated cells and metabolites, offer a safer, more stable alternative to probiotics, with immunomodulatory and anti-inflammatory effects that support gut barrier restoration and microbiota balance, making them promising for IBD management. Their ability to improve mucosal health without the risks associated with live microbial therapies makes them attractive candidates for IBD management [15].

To better understand how probiotics and their derivatives influence the epithelial barrier in the context of IBD, we developed an in vitro model of intestinal inflammation by exposing HT-29 cells to fecal supernatants derived from UC patients during active disease flare-ups. Using this system, we investigated the modulatory effects of different preparations of *Lactobacillus helveticus* SBT2171, namely live bacteria, cell-free supernatant, and supernatant containing bacterial debris, on epithelial gene expression. By focusing on *Claudin1* and *MUC2*, two key markers of tight junction integrity and mucosal protection, our study aims to elucidate the potential of probiotic-based interventions to restore epithelial homeostasis under inflammatory conditions.

## Methods and materials

### Sample collection and preparation

Between March and September 2022, stool samples were obtained from patients diagnosed with IBD at the Liver and Gastroenterology Clinic of Taleghani Hospital, following ethical guidelines and with informed consent obtained from all participants (The study was approved by the local ethics committee under the code: IR.SBMU.RIGLD.REC.1401.007). Participants completed a screening questionnaire collecting demographic data (e.g., age, personal and family history of autoimmune diseases or malignancies, history of intestinal surgery, and alcohol and tobacco use). Patients with cancer, autoimmune diseases other than IBD, gastrointestinal surgery, or chronic alcohol/tobacco use were excluded (Table 1).

**Table 1:** Clinical and demographic characteristics of ulcerative colitis patients providing fecal supernatants

Characteristic	Patient 1	Patient 2	Patient 3
Disease type	Ulcerative colitis	Ulcerative colitis	Ulcerative colitis
Disease duration	~ 1.5 years	~ 6 months	~ 5 years
Disease phase (based on clinical evaluation)	Active flare	Active flare	Active flare
Smoking status	No	No	No
Alcohol consumption	No	No	No
Current UC medication	Mesalazine (5-ASA)	Mesalazine (5-ASA)	Mesalazine (5-ASA)
Medication dose & duration	6 tablets/day, 1.5 years	6 tablets/day, 6 months	6 tablets/day, 5 years
Other medications	None	None	None
History of colon resection	No	No	No
Other gastrointestinal diseases	Reflux	None	None
Other autoimmune diseases	No	No	No
Family history of cancer	Yes	No	No

Fecal supernatant (FS) was prepared from the IBD patient confirmed to be in the active disease phase based on clinical evaluation at the time of sample collection. Under sterile conditions, stool sample was collected and transferred into a 10 mL Falcon tube. Phosphate-buffered saline (PBS) was added at twice the stool volume, and the mixture was vortexed to ensure uniform suspension. After centrifugation at 40,000 × g for 2 h at 4 °C, the supernatant was filtered through a 0.22 μm sterile filter (Jet Biofil, China), aliquoted, and stored at − 80 °C until use. For in vitro inflammation assays, serial dilutions of the FS were prepared by adding 100 µL of the extract to 900 µL PBS, followed by successive 1:10 dilutions across ten tubes to obtain a range of concentrations for cell treatment.

### Cell culture (HT-29 Cells)

The human colorectal adenocarcinoma cell line HT-29 (ATCC HTB-38) was obtained from the Iranian Biological Resource Center (IBRC). Cells were cultured in Dulbecco’s Modified Eagle Medium (DMEM) (Biosera, France) supplemented with 10% fetal bovine serum (FBS) (Gibco, USA), 1% penicillin-streptomycin (Biosera, France), glutamine (GlutaMAX, USA), non-essential amino acids (Sigma-Aldrich, USA) and maintained at 37 °C in a humidified incubator with 5% CO₂. Cells were passaged 3–5 times before use in experiments.

### Inflammation induction in HT-29 Cells

HT-29 cells were first detached from the culture flask using trypsin-EDTA (Gibco, USA) and subsequently resuspended in complete DMEM. The cells were then seeded into two plates and treated with various concentrations of FS. Each plate included duplicate wells for each concentration, as well as control wells containing only PBS. Plates were incubated at 37 °C with 5% CO₂ for the 24 and 48 h.

After incubation, supernatants were collected and stored in labeled microtubes. Cells were gently washed with PBS, followed by treatment with trypsin. After detachment, medium was added to each well, and the cell suspensions were collected by pipetting. Samples were centrifuged at 1200 × g for 5 min. Supernatants were discarded, and the cell pellets were stored at − 80 °C for subsequent RNA extraction.

### Bacterial strain and culture conditions

The active strain of *Lactobacillus helveticus* SBT2171 was obtained from the IBRC as a cultured plate. For propagation, the bacteria were grown in de Man, Rogosa, and Sharpe (MRS) medium prepared from MRS agar powder (Sigma-Aldrich, USA). Strain characterization was confirmed through microscopic observation and Gram staining, which verified the typical cell morphology and Gram-positive nature of the bacterium. To promote optimal growth, the strain was subsequently cultured in MRS broth and incubated anaerobically in an anaerobic jar (Anoxomat^®^ AN2CTS) at 37 °C for 48 h.

### Preparation of bacterial components

The strain was then harvested by centrifugation at 6000 × g for 10 min. The bacterial pellet was washed twice with 1 mL sterile PBS and resuspended in PBS. The optical density (OD) at 600 nm was adjusted to match the 0.5 McFarland standard (OD₆₀₀ = 0.09) by dilution with PBS, corresponding to an approximate concentration of 1.5 × 10⁸ CFU/mL. This standardized suspension was used to calculate the multiplicity of infection (MOI) for treatments involving live bacteria and both types of bacterial extracts.

To prepare the extracts, 1 mL of the adjusted bacterial suspension was transferred into two sterile microtubes. The samples were then subjected to heat-shock inactivation by incubating in a 100 °C water bath for 10 min, followed by cooling on ice for a few minutes. This heat-shock cycle was repeated two more times, for a total of three cycles. One microtube, containing the whole heat-killed suspension, was designated as the bacterial extract with debris. The second microtube was centrifuged (6000 × g, 10 min), and the supernatant was collected as the cell-free bacterial extract.

### Treatment of inflamed HT-29 Cells

First, the culture medium was replaced with antibiotic-free medium. Then, HT-29 cells were harvested by trypsinization, counted, and seeded into four plates, with two plates designated for 24-hour incubation and two for 48-hour incubation; all conditions were performed in duplicates. Based on previous experiments, the concentration of FS inducing inflammation was selected. Accordingly, FS from IBD patients was added to all wells except controls. After 24 h of incubation, selected wells were treated with *Lactobacillus helveticus* at MOI 10 and its two bacterial extracts (supernatant and debris-containing supernatant). Following 24 and 48 h of treatment, cells and supernatants were collected from each well and stored at − 70 °C for RNA extraction. These procedures were repeated for samples from all of the three patients.

### RNA extraction and cDNA synthesis

Total RNA was extracted from HT-29 cells using the BioBasic Total RNA Extraction Kit (BioBasic Inc., Canada) according to the manufacturer’s instructions. Complementary DNA (cDNA) synthesis was performed using Addscript cDNA synthesis kit (Addbio, South Korea) following the provided protocol.

### Gene expression analysis by real-time PCR

The mRNA expression levels of *MUC2*, *Claudin-1*, and *IL-8* were quantified using SYBR Green-based real-time PCR. *Glyceraldehyde-3-phosphate dehydrogenase* (*GAPDH*) was used as the internal control. To assess inflammation in HT-29 cells, the expression of *IL-8* was measured in wells treated with various dilutions of stool extract, including wells treated with undiluted extract.

Primers for *IL-8*, *MUC2*, and *Claudin-1* were designed and then validated using Primer-BLAST (NCBI) to ensure specificity. Their secondary structures and potential dimers were analyzed using OligoAnalyzer. Reaction mixtures were prepared according to the number of samples (X) using the following proportions for a total volume of 10 µL per reaction: 3.5 × X µL of distilled water, 0.25 × X µL of forward primer, 0.25 × X µL of reverse primer, and 5 × X µL of SYBR Green Master mix 2X (Amplicon, Denmark).

Quantitative PCR reactions were run using the Rotor-Gene Q real-time PCR system (Qiagen, Germany). All reactions were performed in duplicates to ensure reproducibility and accuracy. The thermal cycling protocol included an initial denaturation step at 95 °C for 5 min, followed by 40 cycles of denaturation at 95 °C for 20 s, annealing at 57 °C (for *IL-8*) or 58 °C (for *MUC2* and *Claudin-1*) for 30 s, and extension at 72 °C for 20 s (Table 2).

**Table 2:** Primer sequences used for qRT-PCR analysis of *Claudin1*, *MUC2*, *GAPDH*, and *IL-8*. F: Forward Primer; R: Reverse Primer; bp: base pairs; Tm: Melting Temperature (°C); MUC2: Mucin 2; GAPDH: Glyceraldehyde-3-Phosphate Dehydrogenase

Gene	Primer Sequence	bp	Product length	Tm
*Claudin1*	*F: CCC TAT GAC CCC AGT CAA TG* *R: ACC TCC CAG AAG GCA GAGA*	20 19	88	58 °C
*MUC2*	*F: CCC AGA AGG CAC CGT ATA TG* *R: TAC AGA CAC ACT GCT CGC A*	20 19	134	58 °C
*GAPDH*	*F: CTC AAG ATC ATC AGC AAT GCC T* *R: ACA GTC TTC TGG GTG GCA GT*	22 20	134	58 °C
*IL-8*	*F: ACC GGA AGG AAC CAT CTC AC* *R: GGC AAA ACT GCA CCT TCA CAC*	20 21	108	57 °C

### Statistical analysis

Relative gene expression levels were calculated using the 2^^−ΔΔCt^ method. Statistical analyses were conducted using GraphPad Prism version 10 (GraphPad Software, USA). Prior to comparison, data were tested for normal distribution. For normally distributed data, comparisons between two groups were performed using the ordinary one-way ANOVA. Results are presented as fold change ± 95% confidence interval (CI) of difference, and a p-value of < 0.05 was considered statistically significant.

## Results

### Fecal supernatant was successfully prepared from selected IBD patients

Fecal samples were collected from patients attending the Liver and Gastroenterology Clinic at Taleghani Hospital. Samples meeting the inclusion criteria, based on questionnaire responses and the absence of recent antibiotic use, were selected for the study. Ultimately, fecal samples from three individuals diagnosed with ulcerative colitis and experiencing sustained disease flares were chosen for further analysis. Following transport to the laboratory, FS were prepared by centrifugation and filtration of the supernatant using a standardized protocol.

### Undiluted fecal supernatant induces robust *IL-8* expression in HT-29 cells

**Fig. 1:**
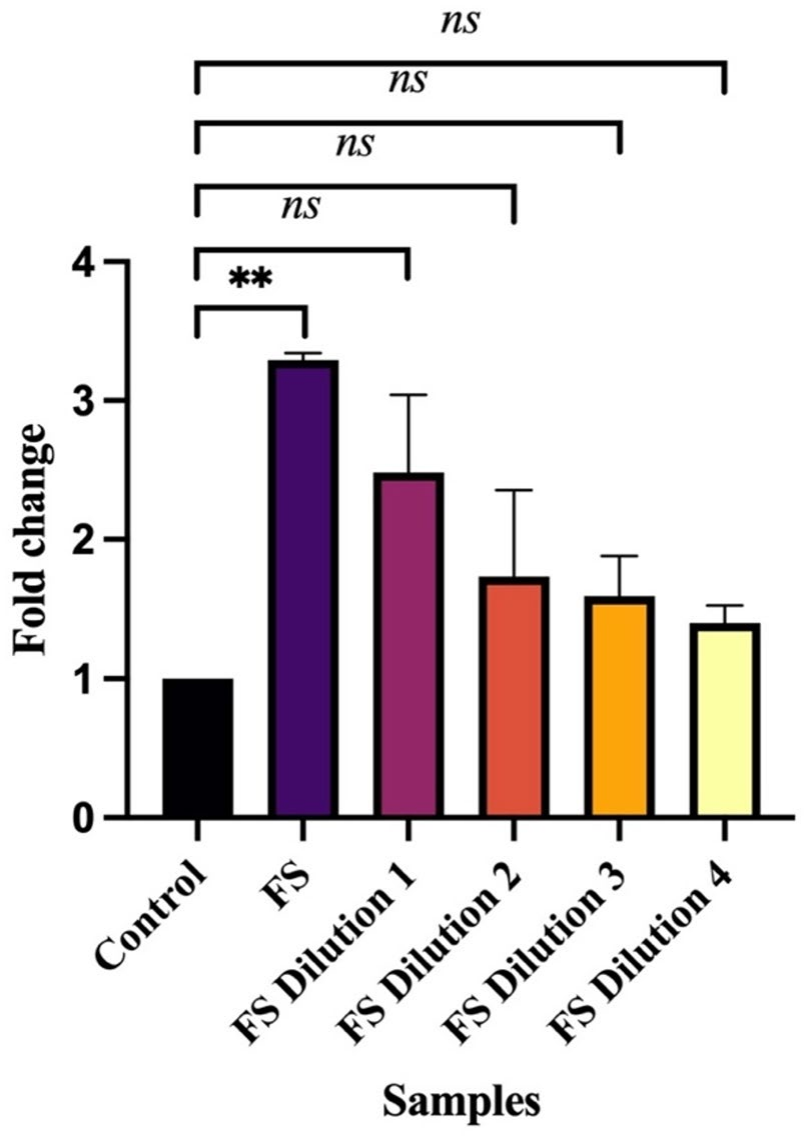
Effect of fecal supernatant dilution on *IL-8* mRNA expression in HT-29 cells. *IL-8* expression was significantly increased following treatment with undiluted fecal supernatant compared with control cells. In contrast, none of the serially diluted fecal supernatant treatments resulted in a statistically significant change in *IL-8* expression relative to the control. Data are presented as fold change. (** represents p-value of < 0.01); Error bars represent standard error of mean (SEM); ns: not significant

To determine the inflammatory potential of FS, HT-29 cells were treated with either undiluted FS or serial dilutions, and the expression of the pro-inflammatory cytokine *IL-8* was quantified via real-time PCR. Treatment with undiluted FS resulted in a significant upregulation of *IL-8* mRNA expression (~ 3.5 fold vs. control; *p* = 0.0085), indicating a strong inflammatory response. In contrast, none of the diluted FS treatments (dilutions 1–4) elicited statistically significant changes in *IL-8* expression compared to the control (*p* > 0.05), suggesting a loss of pro-inflammatory effect upon dilution. Based on these findings, undiluted FS (500 µg/mL) was selected as the standard concentration for subsequent induction of inflammation in HT-29 cells (Fig 1).

### Fecal supernatants from IBD patients differentially modulate *MUC2* and *Claudin1* expression

To investigate the effects of IBD-derived inflammatory environments on intestinal epithelial barrier markers, HT-29 cells were exposed to FS from three individual UC patients. Gene expression of the tight junction marker *Claudin1* and the secretory mucin gene *MUC2* was assessed by qPCR.

**Fig. 2:**
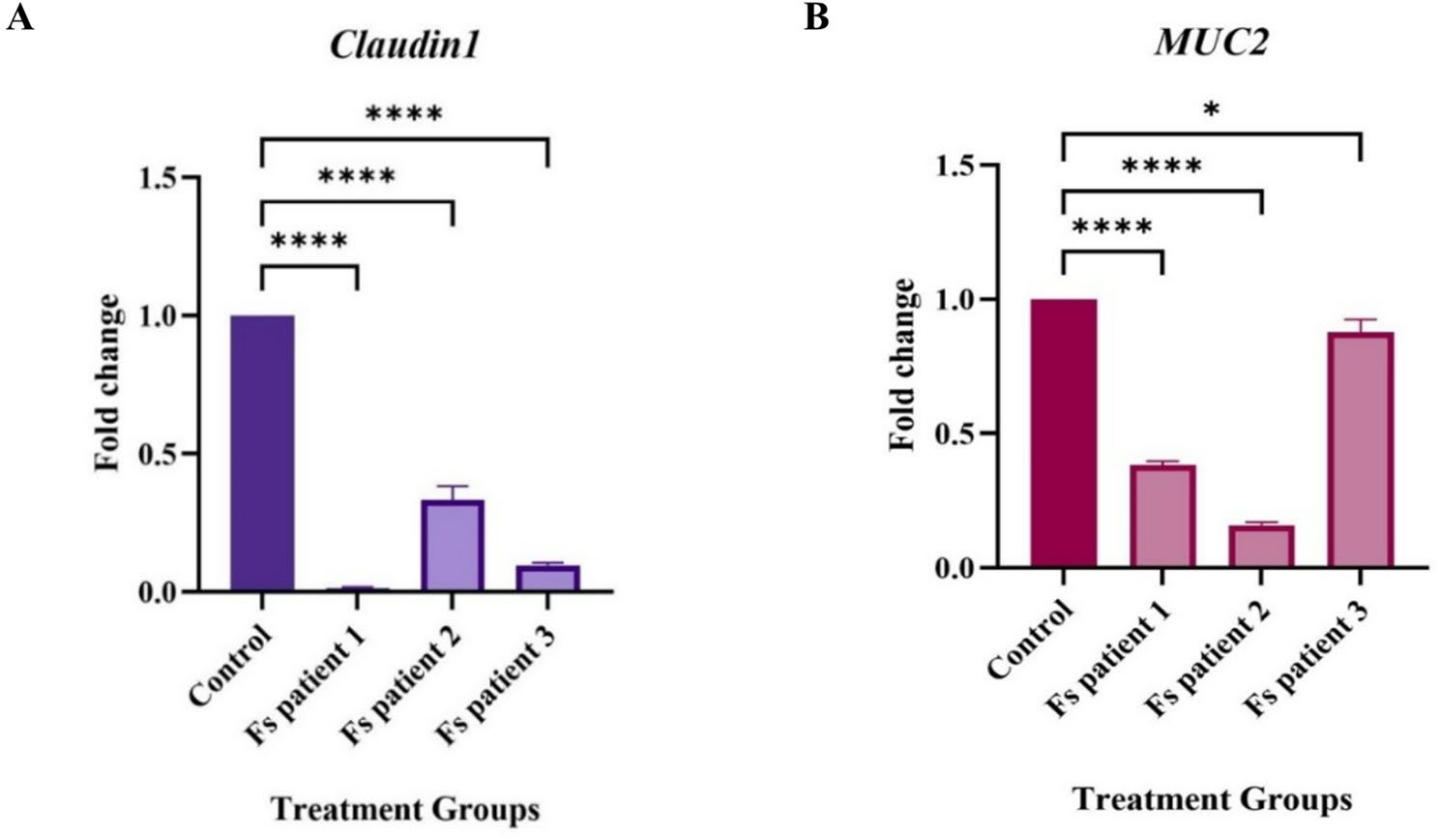
*Claudin1* and *MUC2* expression in HT-29 cells exposed to FS from UC patients. **A ***Claudin1* expression was significantly downregulated in HT-29 cells treated with FS from all three UC patients, as assessed by qPCR. FS from Patient 1 caused near-complete suppression (Fold change ≈ -0.02; *p* < 0.0001), while FS from Patients 2 and 3 also induced marked reductions (Fold change ≈ -0.45 and − 0.15, respectively; *p* < 0.0001). These results indicate a consistent impairment of tight junction gene expression across patient samples. **B ***MUC2* expression showed variable responses to FS treatment. FS from Patients 1 and 2 significantly reduced *MUC2* levels (Fold change ≈ -0.35 and − 0.15, respectively; *p* < 0.0001), whereas FS from Patient 3 slightly decreased *MUC2* expression (*p* = 0.02). These findings highlight a patient-specific modulation of mucin gene expression in response to UC-associated inflammatory stimuli (* and **** represent p-values of < 0.05 and < 0.0001, respectively); Error bars represent standard error of mean (SEM). MUC2: Mucin 2; FS: Fecal Supernatant

*Claudin1* expression was uniformly and significantly downregulated by FS from all three patients. Compared to untreated control cells, FS from Patient 1 nearly abolished *Claudin1* expression (Fold change ≈ -0.02; CI: 0.88–1.08; *p* < 0.0001), while FS from Patient 2 and Patient 3 also induced strong suppression (Fold change ≈ -0.45 and − 0.15; respectively; CI: 0.73–0.94 and 0.01-1.0, respectively; *p* < 0.0001 for both). These results indicate that UC patient-derived FS consistently impairs epithelial tight junction gene expression, regardless of patient-specific variation (Fig. 2A).

In contrast, *MUC2* expression showed a more heterogeneous pattern. FS from Patient 1 and Patient 2 significantly reduced *MUC2* expression (Fold change ≈ -0.35 and − 0.15 vs. control; CI: 0.51–0.72, 0.73–0.94, respectively; *p* < 0.0001). However, in cells treated with FS from Patient 3, *MUC2* expression was relatively preserved and slightly decreased (Fold change ≈ 0.9; CI: 0.01–0.22; *p* = 0.02). These findings highlight a patient-specific modulation of mucin gene expression by UC-associated inflammatory stimuli, in contrast to the uniform suppression observed for *Claudin1* (Fig. 2B).

### *Claudin1* expression in inflamed HT-29 cells is differentially regulated by *L. helveticus*

To assess whether *Lactobacillus helveticus* could modulate epithelial barrier function under inflammatory conditions, pre-inflamed HT-29 cells with FS from the three UC patients were treated with live *L. helveticus* (MOI10), bacterial supernatant, or supernatant combined with bacterial debris. *Claudin1* expression was analyzed at 24 and 48 h post-treatment.

#### Patient 1

**Fig. 3:**
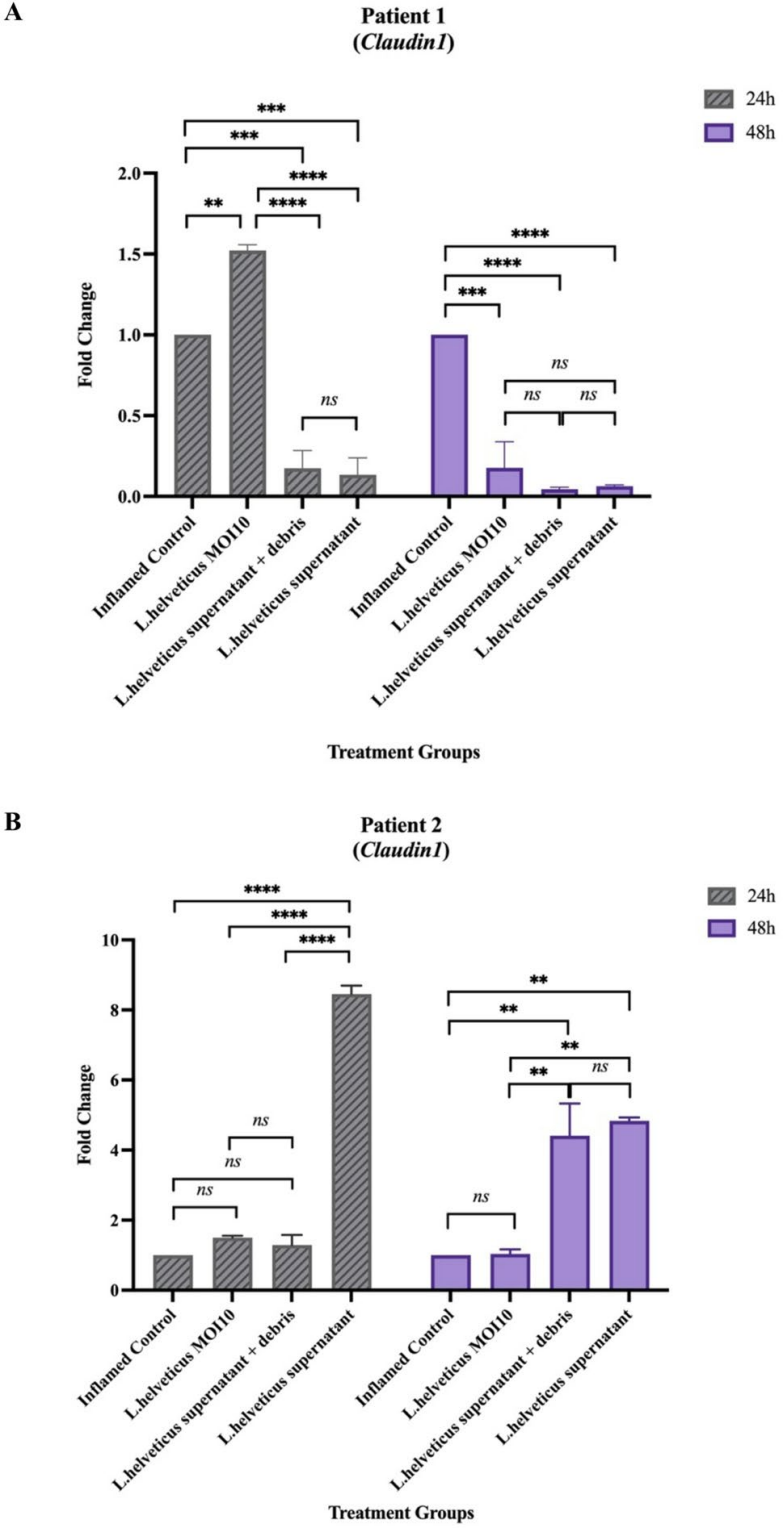

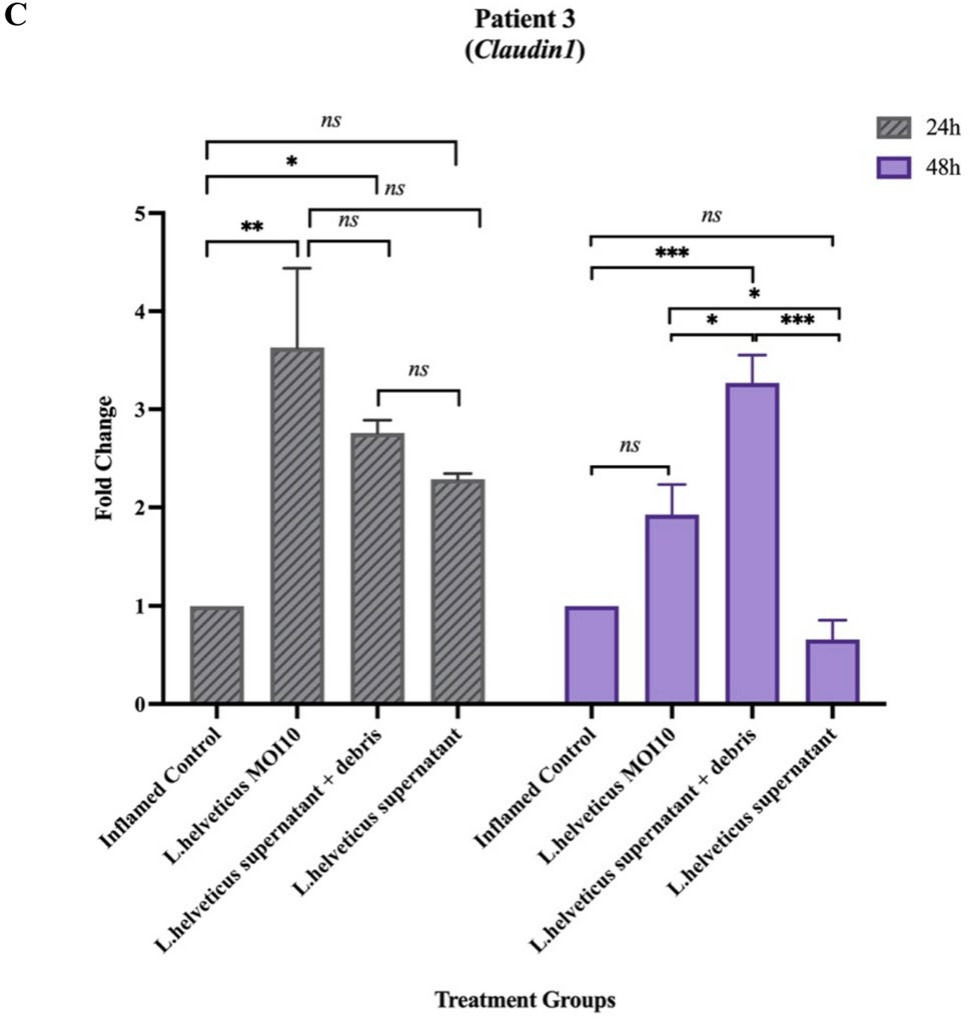
Modulation of *Claudin1* expression in pre-inflamed HT-29 cells treated with live Lactobacillus helveticus (MOI10), bacterial supernatant, or supernatant plus debris from three UC patients. **A** For Patient 1, Live *L. helveticus* transiently increased *Claudin1* at 24 h (~ 1.6-fold; *p* < 0.001), but expression decreased below control by 48 h (*p* < 0.0001). Supernatant treatments had no effect, and live bacteria induced significantly higher *Claudin1* than both supernatant conditions at 24 and 48 h. **B** In patient 2, *L. helveticus* supernatant strongly increased *Claudin1* at 24 h (~ 8.5-fold; *p* < 0.0001), exceeding live bacteria and debris-containing supernatant. Elevated expression persisted at 48 h with supernatant treatments (*p* < 0.01), while live bacteria showed no significant effect, indicating soluble factor–driven regulation. **C** For Patient 3, all treatments elevated *Claudin1* at 24 h, with live bacteria showing the strongest effect (Fold change ≈ 3.6; *p* < 0.01); expression remained elevated at 48 h in live bacteria and debris groups. These results indicate patient-specific differences in the mechanisms by which *L. helveticus* and its components modulate epithelial barrier gene expression under inflammatory conditions (*, **, and *** represent p-values of < 0.05, < 0.01, and < 0.001, respectively); Error bars represent standard error of mean (SEM). MOI: Multiplicity of Infection; ns: non-significant

Live *L. helveticus* significantly upregulated *Claudin1* expression at 24 h (fold change ≈ 1.6; CI: −0.83 to − 0.2; *p* < 0.001). However, this effect was lost by 48 h, with expression falling significantly below control levels (*p* < 0.0001). Neither bacterial supernatant nor supernatant with debris restored *Claudin1* expression at either time point. Importantly, *Claudin1* expression in the live bacteria–treated group was significantly higher than in both supernatant-treated conditions at 24 and 48 h (*–**), whereas no significant differences were observed between supernatant with debris and supernatant alone. These findings indicate a transient, live bacteria–dependent regulation of *Claudin1* expression (Fig. 3A).

#### Patient 2

In contrast, *L. helveticus* supernatant induced a significant increase in *Claudin1* expression at 24 h (fold change ≈ 8.5; CI: −8.22 to − 0.68; *p* < 0.0001), significantly exceeding the effects of both live bacteria and supernatant with debris (****). This elevated expression persisted at 48 h in cells treated with supernatant and supernatant with debris (*p* < 0.01), whereas live bacteria produced only a modest, non-significant change. At both 24 and 48 h, *Claudin1* expression in the live bacteria–treated group was significantly lower than in the supernatant-treated conditions. Notably, no significant difference was observed between supernatant with debris and supernatant alone at 48 h, indicating comparable effects of these two treatments at this later time point. Collectively, these data indicate that soluble bacterial factors, rather than live bacteria, are the primary drivers of *Claudin1* upregulation in this patient’s inflammatory context (Fig. 3B).

#### Patient 3

All three treatments increased *Claudin1* expression at 24 h, with the strongest induction observed following exposure to live *L. helveticus* (fold change ≈ 3.6; CI: −4.3 to − 0.95; *p* < 0.01). At this time point, *Claudin1* expression in the live bacteria–treated group was slightly higher than in cells treated with supernatant with debris (ns), also no significant difference was detected between live bacteria and supernatant alone or between the two supernatant-based treatments. At 48 h, *Claudin1* expression remained elevated in the live bacteria and supernatant with debris groups (fold change ≈ 1.9 and 3.2, respectively), with supernatant with debris inducing significantly higher expression than both live bacteria and supernatant alone (***). Live bacteria and supernatant alone showed only a marginal difference at 48 h (*). Collectively, these findings indicate a time-dependent shift in *Claudin1* regulation in Patient 3, with early responses favoring live bacteria and later responses dominated by debris-associated soluble factors (Fig. 3C).

### *MUC2* induction by *L. helveticus* depends on both bacterial form and inflammatory context

The effect of *L. helveticus* on mucin gene expression was also evaluated following inflammation induction. HT-29 cells were exposed to FS from the three UC patients and subsequently treated with live bacteria, bacterial supernatant, or supernatant plus debris. *MUC2* expression was measured at 24- and 48-hours post-treatment.

#### Patient 1

**Fig. 4:**
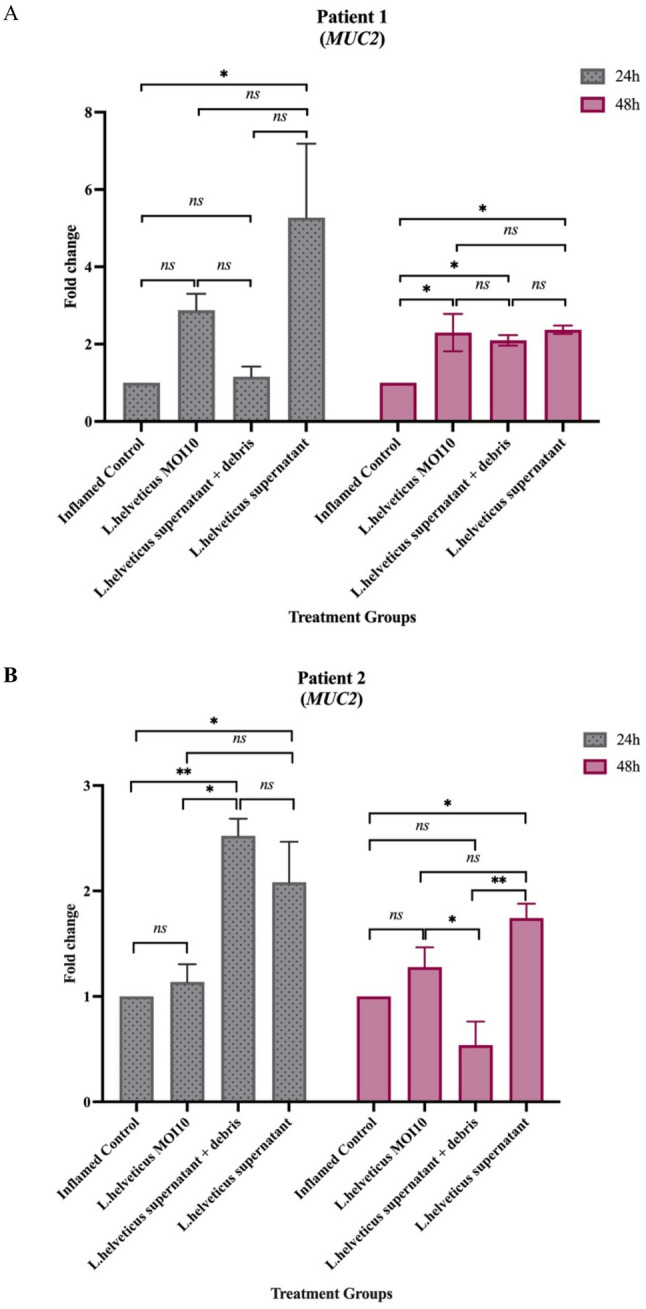

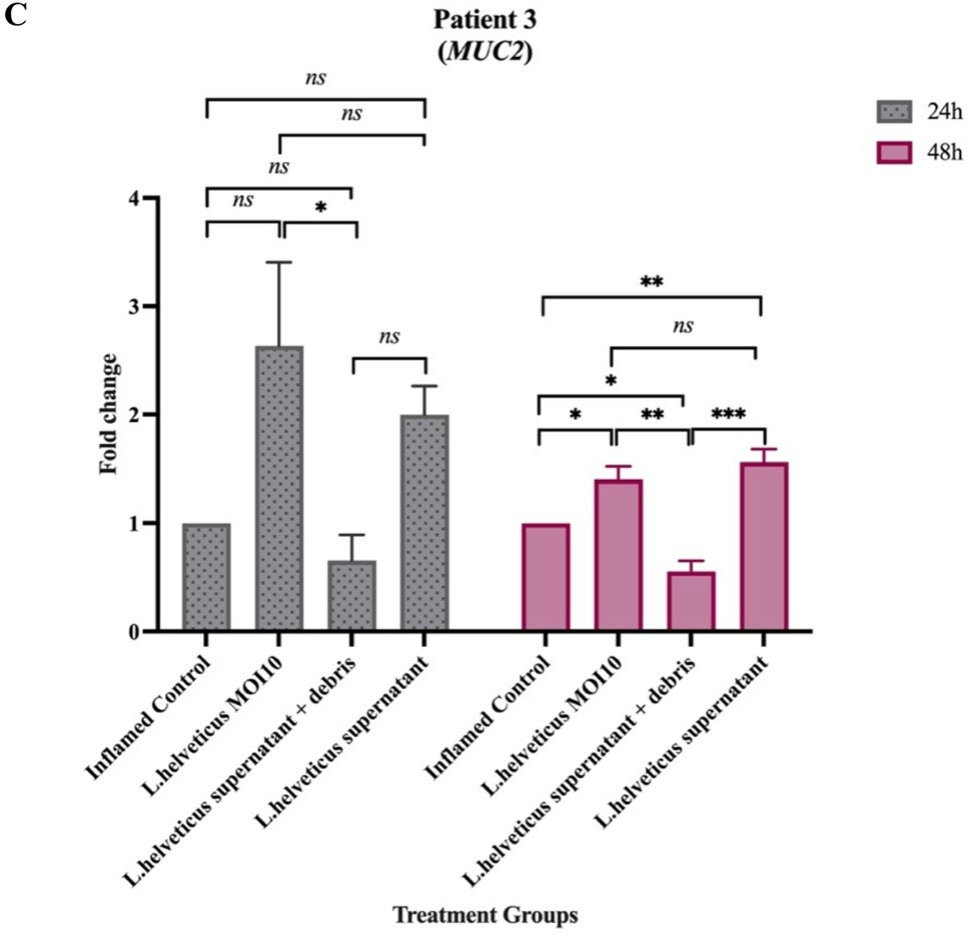
Effects of live *Lactobacillus helveticus*, bacterial supernatant, and supernatant plus debris on *MUC2* expression in pre-inflamed HT-29 cells treated with FS from three UC patients. **A** For Patient 1, bacterial supernatant significantly upregulated *MUC2* at 24 h (Fold change ≈ 6; *p* < 0.05), with live bacteria and debris also increasing expression; all treatments sustained elevated levels at 48 h (Fold change ≈ 2.2–2.4; *p* < 0.05). **B** In Patient 2, supernatant and debris significantly enhanced *MUC2* at 24 h (Fold change ≈ 2.0 and 2.5; *p* < 0.05 and *p* < 0.01), with supernatant maintaining highest expression at 48 h (Fold change ≈ 2.6; *p* < 0.05). **C** For Patient 3, At 24 h, *MUC2* levels were similar across treatments, with live bacteria higher than supernatant + debris (*p* < 0.05). At 48 h, live bacteria and supernatant maintained elevated *MUC2*, while debris attenuated induction, indicating sustained expression is driven by live bacteria and soluble factors. These findings demonstrate patient-dependent modulation of mucin gene expression by *L. helveticus* and its components under inflammatory conditions (* and ** represent p-values of < 0.05 and < 0.01, respectively); Error bars represent standard error of mean (SEM). MOI: Multiplicity of Infection; ns: non-significant

The supernatant significantly upregulated *MUC2* expression at 24 h (Fold change ≈ 6 vs. control; CI: -8.29 to -0.24; *p* < 0.05). Live bacteria and debris treatments also increased *MUC2* (Fold change ≈ 2.7 and 1.6; CI: -5.9 to 2.14 and − 4.17 to 3.86, respectively), though without significant differences between them. At 48 h, all treatments maintained significantly elevated *MUC2* expression compared to control (*p* < 0.05), with similar fold changes (Fold change ≈ 2.2–2.4) (Fig. 4.A).

#### Patient 2

At 24 h, both *L. helveticus*–derived supernatant (*p* < 0.05) and supernatant + debris (*p* < 0.01) significantly increased *MUC2* expression compared with live bacteria, which showed no significant effect. The supernatant + debris group induced slightly higher *MUC2* levels than supernatant alone but it was not significant. At 48 h, *MUC2* expression was significantly higher in the supernatant group compared with live bacteria (*p* < 0.05), whereas the supernatant + debris group showed reduced expression and did not differ significantly from live bacteria (ns). Direct comparison revealed significantly higher *MUC2* levels in the supernatant group than in the supernatant + debris group (*p* < 0.01). Overall, these findings indicate that while both cell-free preparations enhance *MUC2* at early time points, sustained induction is driven primarily by the soluble supernatant fraction (Fig. 4B).

#### Patient 3

At 24 h, live *L. helveticus*, supernatant + debris, and supernatant alone showed no significant differences in *MUC2* expression, although live bacteria induced significantly higher levels than supernatant + debris (*p* < 0.05). At 48 h, live bacteria significantly increased *MUC2* expression compared with supernatant + debris (< 0.01), while no significant difference was observed between live bacteria and supernatant alone (ns). Supernatant treatment produced the highest *MUC2* levels and was significantly greater than supernatant + debris (*p* < 0.001). Overall, these results indicate that bacterial debris attenuates *MUC2* induction, whereas live bacteria and soluble components promote sustained expression in Patient 3 (Fig. 4C).

## Discussion

In this study, we established an in vitro model of intestinal inflammation by exposing HT-29 cells to fecal supernatants derived from patients with ulcerative colitis during active flare-ups. This approach aimed to replicate the complex, pro-inflammatory intestinal environment characteristic of IBD. Following inflammation induction, we investigated the effects of different components of the probiotic *Lactobacillus helveticus* SBT271, specifically live bacterial cells, cell-free supernatant, and supernatant containing bacterial debris, on restoring intestinal barrier function. By assessing the expression of genes associated with epithelial integrity, we evaluated how these probiotic fractions modulate inflammation-induced disruptions in epithelial homeostasis.

FS from individuals with IBD exhibit distinct features compared to those from healthy controls, largely due to elevated proteolytic activity originating from both host and microbial serine and metalloproteases. This heightened activity has been consistently observed in IBD patients and is supported by metagenomic, proteomic, and metabolomic analyses [16]. To replicate IBD-like inflammation, we used patient-derived FS instead of standard inducers such as lipopolysaccharide (LPS). Unlike LPS, FS encompasses a wide array of microbial products, metabolites, and inflammatory mediators that vary among individuals, better reflecting the heterogeneous and patient-specific inflammatory milieu seen in vivo [17]. his strategy introduces biological variability that closely mirrors the complexity of IBD pathogenesis.

Exposure of HT-29 cells to UC-derived FS consistently led to downregulation of *Claudin1* and *MUC2*, two genes essential for maintaining epithelial barrier integrity. All three FS samples induced significant suppression of *Claudin1* expression. Claudins are essential structural proteins of the tight junction complex and are fundamental to regulating paracellular permeability and maintaining barrier cohesion [18]. Despite differences in disease presentation and microbiota composition among patients, the consistent downregulation suggests a shared pathogenic mechanism targeting junctional stability. The marked reduction of *Claudin1* in response to Patient 1’s FS (Fold change ≈ -0.02) underscores the potent barrier-disruptive capacity of IBD-associated luminal contents, likely due to the presence of proteases, cytokines, or other bioactive molecules.

In contrast, *MUC2* expression showed more variability. Secreted by goblet cells, *MUC2* forms the intestinal mucus layer and protects epithelial cells from luminal microbes [19, 20]. While FS from Patients 1 and 2 led to marked reductions in *MUC2* expression, Patient 3’s FS had a milder effect, with only a modest but statistically significant decrease. This variation likely reflects differences in microbial communities or inflammatory mediators, suggesting that mucin gene regulation is more context-dependent compared to tight junction components. The preparation containing microbial debris carries components of the bacterial cell wall, including lipoteichoic acids, peptidoglycan fragments, and other microbe-associated molecular patterns (MAMPs). Under inflammatory conditions, these MAMPs may overactivate epithelial pattern recognition receptors (such as TLR2 and NOD2), triggering a pro-inflammatory response that impairs goblet cell activity and *MUC2* production [21]. This mechanism could account for the inhibitory effect seen in patient 3.

Our findings support existing literature suggesting that UC fecal supernatants contain bioactive factors capable of modulating epithelial and immune responses. For instance, Dabek et al. [22] showed that intracolonic infusion of UC FS in mice increased intestinal permeability and neutrophil infiltration via Cathepsin G-mediated activation of protease-activated receptor-4 (PAR4). Although our system is in vitro, we observed similar indicators of barrier disruption and inflammation, including elevated *IL-8* expression and *Claudin1* suppression. The patient-specific variability in *MUC2* expression observed aligns with Dabek et al.‘s findings of heterogeneity in microbial and protease profiles, highlighting the individualized nature of host–microbiota interactions in UC. In contrast, Crohn’s disease FS appears to act through different mechanisms. Gorreja et al. [23] reported that CD FS induced IL-10 and IL-1RA in M2 macrophages while promoting IL-6 and MCP-1 secretion in fibroblasts. This suggests that whereas UC FS primarily compromises epithelial integrity, CD FS exerts broader effects on immune and stromal cells. Our epithelial-focused findings further reinforce this dichotomy, with UC FS exhibiting strong pro-inflammatory effects on epithelial gene expression.

Probiotics, including *Lactobacillus* species, have shown promise in supporting intestinal homeostasis by enhancing tight junctions, increasing mucin expression, modulating immune responses, and preventing pathogen adhesion [11, 24]. Postbiotics, non-viable microbial components such as inactivated cells, metabolites, and bacterial fractions, represent a stable and potentially safer alternative. Their immunomodulatory and anti-inflammatory properties position them as viable candidates for gut barrier repair and microbiota rebalancing in IBD therapy [15].*L. helveticus* is known for typical probiotic traits, including epithelial adherence, resistance to gastric transit, and antagonism against pathogens [25]. Accordingly, we evaluated the effects of live bacteria, supernatant, and debris-containing supernatant on FS-inflamed HT-29 cells. An MOI of 10 was used as the standard screening dose to assess probiotic strain activity, as it produced a clear effect without overwhelming the cells [26]. Our data indicate that the efficacy of each treatment varied depending on both the bacterial preparation and the individual patient’s FS.

Following treatment with FS from Patient 1, *Claudin-1* expression was almost completely suppressed, indicating severe disruption of the epithelial barrier. Patient 1 had been diagnosed with UC for about 1 year and half and had been in the flare phase for about a month at the time the fecal supernatants were obtained. This relatively recent flare may explain the profound barrier disruption observed. Subsequent treatment with *Lactobacillus helveticus* showed that only the live bacteria could restore *Claudin-1* expression, with a notable increase observed at 24 h. However, this effect was not sustained at 48 h, suggesting a transient impact of live bacteria under continued inflammatory stress. Neither the bacterial supernatant nor the supernatant with debris induced any measurable improvement, highlighting the necessity of direct interaction with live bacteria for barrier repair in this patient, albeit with limited durability. Treatment with FS from Patient 2 caused a moderate reduction in *Claudin-1* levels. Patient 2 had a longer disease duration of 5 years and had been in the flare phase for 4 months at the time the fecal supernatants were obtained, indicating a more chronic inflammatory state. Interestingly, all forms of *L. helveticus*, including live bacteria, supernatant with debris, and supernatant alone, led to significant upregulation of *Claudin-1*, especially at 48 h. Among them, the bacterial supernatant induced the strongest and most sustained response across both time points. In contrast, live bacteria and debris had limited effects. These findings suggest that in patient 2, soluble bioactive factors secreted by *L. helveticus* were more effective than direct bacterial interaction, indicating a broader sensitivity to probiotic-derived metabolites. FS from Patient 3 led to a severe reduction in *Claudin-1* expression, though not as extreme as in patient 1. Notably, Patient 3 was diagnosed with UC relatively recently (6 months) but had been in the flare phase for 5 months at the time the fecal supernatants were obtained, indicating a persistent active inflammation despite the shorter overall disease duration. Upon secondary treatment, all conditions improved *Claudin-1* levels at 24 h, with the live bacteria and debris-containing supernatant maintaining elevated expression even at 48 h. The supernatant alone, however, had only a mild effect. This pattern suggests that multiple bacterial components, including structural elements and live microbial activity, contributed to a more durable restoration of the epithelial barrier in this patient. While the precise mediators remain unidentified, the response reflects a more flexible interaction with various forms of *L. helveticus*.

*MUC2* expression, essential for maintaining the intestinal mucus barrier, was differentially affected by FS from these patients. In Patient 1, FS led to a significant reduction in *MUC2*, indicating the presence of mucin-suppressive components. Subsequent treatment with *Lactobacillus helveticus*, whether live bacteria, bacterial supernatant, or supernatant with debris, restored *MUC2* levels, especially at 48 h. The supernatant alone had the strongest early effect at 24 h, suggesting a rapid response to secreted probiotic factors and a generally receptive epithelial environment. Patient 2 showed the most pronounced suppression of *MUC2*, reflecting a more disruptive or inflammatory fecal profile consistent with the chronic flare state. Here, *L. helveticus* again promoted recovery, with the supernatant + debris most effective at 24 h and the supernatant alone maintaining elevated expression at 48 h. Live bacteria had little impact, suggesting that non-living components were the primary mediators of mucin restoration in this context. In Patient 3, FS had little effect on *MUC2*, but *L. helveticus* enhanced expression beyond baseline in most treatments. An exception was observed with the debris-containing supernatant, which significantly reduced *MUC2* expression by 48 h. These findings suggest that bacterial debris may not only lack beneficial effects but could, in some inflammatory contexts, actively suppress epithelial responses, possibly through stress-related mechanisms or altered epithelial signaling. The prolonged flare in patient 3 may have contributed to this heightened sensitivity to certain bacterial components. Also, UC-derived FS contains increased levels of host and microbial proteases, such as neutrophil elastase, cathepsin G, and bacterial serine proteases, which can either directly break down *MUC2* or suppress its transcription via ER stress–related pathways. Differences in protease composition and activity, along with the presence of mucin-degrading bacteria like *Ruminococcus gnavus* or *Akkermansia muciniphila*, may explain the heterogeneous effects of FS on *MUC2* across patients [27].

Our findings align with previous studies highlighting the beneficial effects of *Lactobacillus* species on intestinal barrier function and inflammation. The cell-free supernatant of *L. helveticus* likely harbors bioactive compounds, including short-chain fatty acids (SCFAs), bacteriocin-like peptides, and small peptides derived from proteolysis, which can directly influence epithelial signaling. Among SCFAs, butyrate is particularly effective in promoting *Claudin1* transcription through the activation of AMP-activated protein kinase (AMPK) and histone acetylation pathways, thereby enhancing tight junction assembly [28]. Several investigations have shown that *L. helveticus* and related probiotics can enhance tight junction integrity, modulate cytokine production, and reduce epithelial inflammation in both in vitro and in vivo models. Alipour et al. [29] showed that live *L. helveticus* reduced pathogen adhesion and modulated cytokines in inflamed colonic cells. Jeffrey et al. [30] found that *L. helveticus* supernatants suppressed pro-inflammatory chemokines in HT-29 cells, supporting our results regarding the efficacy of bacterial metabolites. Similarly, Alard et al. [31] highlighted the strain-specific barrier-enhancing effects of *L. helveticus* in acute colitis models. Protective roles of dairy-derived *Lactobacillus* strains were further confirmed by Rezai et al. [32], who demonstrated anti-inflammatory and antioxidant benefits in UC mouse models. De Marco et al. [33] also reported inflammation reduction in epithelial and immune cells via cell-free supernatants. Moreover, Yamashita et al. [34] showed systemic immunomodulation by oral *L. helveticus* in arthritis models, suggesting broader anti-inflammatory properties that may translate to intestinal inflammatory conditions.

Altogether, our results recapitulate key features of epithelial disruption observed during intestinal inflammation and suggest that bacterial-derived components may modulate pathways associated with intestinal barrier function in vitro. However, the effects of *L. helveticus* were not uniform and depended on both the form of bacterial preparation and the inflammatory context. The distinct responses elicited by each FS sample highlight the complexity of host–microbiota interactions and reinforce the need for personalized approaches to microbiota-based therapies in IBD.

This study has several important limitations that should be acknowledged. First, our analyses were limited to transcriptional changes in barrier-associated genes, and we did not assess protein expression or barrier functionality. As a result, changes in *Claudin-1* and *MUC2* mRNA levels cannot be directly equated with restored tight junction assembly or mucus layer integrity. Future studies should therefore incorporate protein-level validation, such as Western blotting or immunofluorescence staining of tight junction and mucin proteins, to confirm whether transcriptional modulation translates into structural barrier recovery. Another limitation is the lack of characterization of the bioactive molecules present in the bacterial supernatants and fecal supernatants. Future work should focus on identifying specific metabolites, peptides, or microbial products responsible for the observed effects using targeted or untargeted metabolomic and proteomic approaches. Such analyses may help distinguish beneficial factors from components that exert neutral or inhibitory effects, particularly in debris-containing preparations. Finally, while the HT-29 cell model provides a controlled epithelial system, it does not capture the complexity of the intestinal environment. Validation of these findings in more physiologically relevant models, such as intestinal organoids, co-culture systems with immune cells, or in vivo colitis models, will be necessary to establish the translational relevance of bacterial-derived interventions and to better understand patient-specific responses.

## Conclusion

The ability of *L. helveticus* to restore *MUC2* and *Claudin1* expression, despite prior suppression by inflammatory FS, highlights both its anti-inflammatory and barrier-reparative potential. Importantly, the culture supernatant exhibited comparable, and in some cases superior, efficacy to live bacteria, suggesting that secreted bioactive compounds (postbiotics) may be a potent therapeutic alternative, especially when live probiotic use is contraindicated.

These findings support the application of *L. helveticus* SBT2171 and its derivatives as promising adjuncts for managing IBD by reinforcing mucosal defenses and alleviating inflammation-induced barrier disruption. Due to the variable responses of probiotics and postbiotics in different FS samples, personalized approaches are necessary to identify the most effective treatment strategies based on patient condition and FS components. Integrating these observations into the broader context of personalized medicine is particularly important, as patient-to-patient heterogeneity in microbiota composition, immune status, and inflammatory mediators can significantly influence therapeutic outcomes. Future in vivo studies are warranted to validate these protective effects and to identify the specific molecular mediators responsible for their action.

## Data Availability

De-identified data supporting the findings of this study are available from the corresponding author upon reasonable request and with approval from the Ethics Committee of Shahid Beheshti University of Medical Sciences (IR.SBMU.RIGLD.REC.1401.007). All other data supporting the results, including experimental protocols and analysis scripts, are available from the authors upon reasonable request.

## Abbreviations

AJ: Adherens Junction
TJ: Tight Junction
IBD: Inflammatory Bowel Disease
CD: Crohn’s Disease
UC: Ulcerative Colitis
FS: Fecal Supernatant
PBS: Phosphate-Buffered Saline
FBS: Fetal Bovine Serum
MRS: Man, Rogosa, and Sharpe (culture medium)
OD: Optical Density
MOI: Multiplicity of Infection
cDNA: Complementary DNA
GAPDH: Glyceraldehyde 3-Phosphate Dehydrogenase
MUC2: Mucin 2
SD: Standard Deviation
LPS: Lipopolysaccharide
PAR4: Protease-Activated Receptor 4
SCFA: Short-chain Fatty Acids
MAMPs: Microbe-associated Molecular Patterns
